# Tumor Progression Locus 2 Differentially Regulates IFNγ and IL-17 Production by Effector CD4^+^ T Cells in a T Cell Transfer Model of Colitis

**DOI:** 10.1371/journal.pone.0119885

**Published:** 2015-03-17

**Authors:** Nicole V. Acuff, Xin Li, Rebecca Kirkland, Tamas Nagy, Wendy T. Watford

**Affiliations:** 1 Department of Infectious Diseases, University of Georgia, 501 DW Brooks Drive, Athens, GA, United States of America; 2 Department of Pathology, University of Georgia, Athens, GA, United States of America; Jackson Laboratory, UNITED STATES

## Abstract

Autoimmune diseases are approaching epidemic levels, estimated to affect 5–8% of the population. A number of autoimmune diseases are believed to be driven by autoreactive T cells, specifically by T helper 1 (Th1) cells and T helper 17 (Th17) cells. One molecule gaining interest as a therapeutic target is the serine-threonine kinase, Tpl2, which promotes expression of proinflammatory mediators. We previously demonstrated that Tpl2 regulates Th1 differentiation, secretion of the inflammatory cytokine IFNγ, and host defense against the intracellular parasite *Toxoplasma gondii*. The goal of this study was to determine whether Tpl2 also regulates Th1 or Th17 differentiation *in vivo* in a model of colitis associated with mixed Th1/Th17 pathology. *In vitro, Tpl2^−/−^* naïve CD4 T cells were significantly impaired in IL-17A secretion under traditional Th17 inducing conditions. Reduced IL-17A secretion correlated with increased expression of FoxP3, a transcription factor known to antagonize RORγt function. In a murine T cell transfer model of colitis, transfer of *Tpl2^−/−^* T cells resulted in reduced proportions of CD4 T cells expressing IFNγ, but not IL-17A, compared to that induced by wild type T cells. Further studies revealed that IL-17A differentiation induced by IL-6 and IL-23, cytokines implicated in driving Th17 differentiation *in vivo*, was unaffected by Tpl2 deficiency. Collectively, these results implicate Tpl2 in TGF-β-induced FoxP3 expression. Additionally, they underscore the contribution of Tpl2 to Th1 immunopathology specifically, which suggests that Tpl2 inhibitors may selectively target Th1-based inflammation.

## Introduction

Tumor progression locus 2, Tpl2 (also known as MAP3K8), is a serine-threonine protein kinase originally described as an oncogene, because its C-terminal truncation promoted tumor growth [1]. Tpl2 is expressed in both innate and adaptive immune cells in diverse tissues, including the spleen, thymus, liver, and lung [1–3]. Activated by toll-like receptors, cytokines, antigen receptors and G protein-coupled receptors [2, 4–10], Tpl2 enhances inflammation by promoting expression of cytokines, chemokines and other inflammatory mediators [5, 6, 8, 11–14]. Many of Tpl2’s functions have been attributed to its activation of the MEK/ERK pathway (reviewed in [3]). Initial characterization of *Tpl2* ^*−/−*^ mice identified major defects in the induction of proinflammatory cytokines, particularly TNFα, by antigen presenting cells that conveyed resistance to endotoxin-induced shock [5]. Because it promotes inflammatory mediators, Tpl2 is being investigated as a therapeutic target for treating autoimmune diseases [15–17].

We previously demonstrated that Tpl2 promotes Th1 differentiation and IFNγ production in response to the intracellular parasite, *Toxoplasma gondii* [8], inhibits T helper 2 (Th2) cell responses during OVA-induced allergic asthma in mice [18] and promotes T helper 17 (Th17) cell secretion of IL-17A *in vitro* [8]. Th17 cells are a distinct lineage of CD4 T cells that produce IL-17A, IL-17F, IL-21, and IL-22 [19–24]. Together, Th17 effector cytokines are required for the clearance of extracellular bacterial and fungal infections, but dysregulated Th17 responses have also been implicated in the development of autoimmune diseases, including multiple sclerosis, rheumatoid arthritis and inflammatory bowel diseases (reviewed in [25]). The importance of Tpl2 in Th17 cell differentiation *in vivo* has not been extensively studied, but Tpl2 is dispensable for driving Th17 differentiation in experimental autoimmune encephalomyelitis (EAE) [26, 27].

In this study, we addressed whether Tpl2 contributes to the development of colitis, an alternative autoimmune disease, in a T cell specific manner. The importance of Tpl2 in certain aspects of inflammatory bowel diseases (IBD), a complex spectrum of autoimmune diseases of the small intestine and colon, has been studied previously. For example, TNF^ΔARE^ mice that express a stabilized TNF transcript and spontaneously develop colitis, showed delayed onset and attenuated progression of IBD when crossed onto the *Tpl2* ^*−/−*^ background [28]. Because colitis in TNF^ΔARE^ mice is due to accumulation of TNF, these results indicate the importance of Tpl2 in transducing TNF signals. Additionally, in a chemically induced model of colitis, dextran sulfate sodium (DSS) damages intestinal epithelial cells and therefore alters barrier function of the intestines, leading to hematochezia, body weight loss, shortening of the intestine, mucosal ulcers, and infiltration of neutrophils. In this innate immune model of colitis, *Tpl2* ^*−/−*^ mice experienced milder colitis compared to wild type mice with reduced production of inflammatory cytokines IL-1α, IL-1β, IL-6, and IL-17, as well as reduced production of the anti-inflammatory cytokine IL-10 [29]. Despite multiple lines of evidence for Tpl2 in various aspects of colitis development, a T cell-intrinsic function for Tpl2 during colitis has not yet been explored.

We first confirmed that *Tpl2* ^*−/−*^ T cells are impaired *in vitro* in the production of IL-17A under the classical Th17 polarizing conditions of IL-6 and TGF-β, and this impairment was associated with elevated expression of FoxP3. In a T cell transfer model of colitis, Tpl2 ablation within the transferred T cell population reduced the proportion of CD4 T cells expressing IFNγ without altering IL-17 expression. Notably, Tpl2 ablation also increased CD4 T cell accumulation in Rag1-deficient recipients *in vivo*. The discrepancy between Tpl2’s regulation of IL-17 production *in vitro* versus *in vivo* was clarified by the finding that IL-17A production was restored to wild type levels in *Tpl2* ^*−/−*^ Th17 cells when the TGF-β concentration was reduced, neutralizing IL-2 antibody was added, or when Th17 cells were alternatively induced by IL-6 and IL-23, all of which failed to induce FoxP3 expression. This study defined a TGF-β- and FoxP3-restricted defect in IL-17A secretion by *Tpl2* ^*−/−*^ T cells. Overall, these findings demonstrate that Tpl2 is dispensable for Th17 differentiation during a T cell transfer model of colitis where IL-6 and IL-23 have a dominant role but underscore the contribution of Tpl2 to Th1 differentiation in this model.

## Materials and Methods

### Ethics Statement

All experiments involving mice were performed according to the University of Georgia guidelines for laboratory animals and were approved by the UGA Institutional Animal Care and Use Committee. The internal IACUC approval number currently is A2012 06-002-Y3-A9.

### Mice

Wild type (C57BL/6) mice were obtained from the Jackson Laboratory (Bar Harbor, Maine). *Tpl2* ^*−/−*^ mice backcrossed onto the C57BL/6 genetic background were kindly provided by Dr. Philip Tsichlis (Tufts University) and Thomas Jefferson University, where the mice were generated. OT-II mice were obtained from the National Institute of Health (NIH), and *Rag1* ^*−/−*^ mice were purchased from Jackson Laboratories. Animals were used at six to twelve weeks of age, and were age- and sex-matched for individual experiments. Animals were bred within the same facility and maintained in sterile microisolator cages.

### Cell sorting

Wild type or *Tpl2* ^*−/−*^ cells from spleens and lymph nodes were disaggregated by pressing through a 70 μm filter, and CD4 T cells were column purified by negative selection using a CD4^+^ T cell isolation kit according to manufacturer’s guidelines (Miltenyi Biotech, Auburn, CA). CD4 T cells were stained for 15 min at 4°C in PBS + 0.5% FBS (Life Technologies, Carlsbad, CA) using anti-mouse antibodies purchased from eBioscience (San Diego, CA): CD16/CD32 (93), CD4 (RM4-5), TCRβ (H57-597), CD25 (PC61.5), CD44 (IM7), CD62L (MGL-14), and CD45RB (C363.16A). Live cells were first gated by excluding propidium iodide positive (PI^+^) cells and then sorted for naïve effectors (CD4^+^CD25^-^CD62L^+^CD44^−^ or CD4^+^CD25^−^CD45RB^hi^) using a Beckman Coulter MoFlo XDP cell sorter.

### Cell culture

Sorted naïve CD4 T cells (CD4^+^CD25^−^CD62L^+^CD44^−^) were enumerated and plated at a concentration of 1x10^6^ cells/ml in cell culture wells with immobilized anti-CD3 and anti-CD28 (5 μg/ml each). Cells were cultured at 37°C and 5% CO_2_ in complete RPMI (RPMI 1640 containing 10% FBS, 100 U/ml penicillin, 100 μg/ml streptomycin, 2 mM L-glutamine (Life Technologies), 0.01 M HEPES (Fisher Scientific, Waltham, MA), and 50 μM 2-mercaptoethanol (Sigma-Aldrich, St. Louis, MO)). Th17 differentiation was induced in the presence of 10 ng/ml IL-6, 5 ng/ml TGF-β, and 10 μg/ml of both anti-IL-4 and anti-IFNγ (BD Biosciences, San Jose, CA) for 3 days, unless otherwise indicated. Additionally, 10 ng/ml IL-1β or 5 μg/ml anti-IL-2 (BD Biosciences) were used in certain polarizing conditions where indicated. Alternatively, for some experiments, Th17 differentiation was induced in the presence of 10 ng/ml IL-6 and 10 ng/ml IL-23 for 3 days in the presence or absence of decreasing concentrations of TGF-β.

Bone marrow-derived dendritic cells (BMDCs) were generated by culture of bone marrow cells from femurs and tibiae of mice. Briefly, bone marrow cells (2x10^6^ cells/ml) were cultured at 37°C and 5% CO_2_ in complete RPMI supplemented with 40 ng/ml GM-CSF (PeproTech, Rocky Hill, NJ). On days 3 and 5, fresh medium equal to half of the initial volume of the culture containing 40 ng/ml GM-CSF was added. On day 7, non-adherent cells were collected, incubated with anti-mouse CD11c labeled microbeads, and CD11c^+^ cells were column purified by positive selection according to manufacturer’s guidelines (Miltenyi Biotech).

For co-culture experiments, 10^4^ wild type BMDCs were incubated with a 10-fold excess of naïve OT-II CD4 T cells (10^5^) which express a transgenic TCR specific for OVA_323–339_ (Peptides International, Louisville, KY). Cells were cultured under neutral (media alone) or Th17 (IL-6 + TGF-β) conditions in 96-well microtiter plates in a volume of 200 μL for 3 days.

### Cytokine Measurements

IL-17A expression, as determined by intracellular staining followed by flow cytometry, was the primary measure of Th17 development. Prior to cell staining, cells were stimulated 4 hours at 37°C with 50 ng/ml PMA (Sigma-Aldrich), 0.5 μg/ml ionomycin (Sigma-Aldrich), and golgi transport inhibitor (BD Biosciences) according to manufacturer’s specifications. The following anti-mouse monoclonal antibodies used were from eBiosciences: CD16/CD32 (93), CD4 (RM4–5), IL-17A (eBio17B7), FoxP3 (FJK-16s), and IFNγ (XMG1.2). Prior to intracellular staining, cells were fixed in either 4% formalin or fixation/permeabilization buffer (eBioscience) and subsequently washed and stained in permeabilization buffer (eBioscience). Samples were run on a BD LSRII flow cytometer and analyzed using FlowJo software (Tree Star, Inc., Ashland, OR). IL-17A, IL-17F, IL-22, IL-2 and IFNγ proteins were measured in supernatants by ELISA (eBiosciences) or Th1/2/17 cytokine bead array (BD Biosciences) according to manufacturer’s guidelines. RNA was isolated from cell pellets on day 3 (unless otherwise indicated) or colon tissue using EZRNA extraction kit (Omega Bio-Tek, Norcross, GA) and converted to cDNA by high capacity cDNA reverse transcription kit (Life Technologies). Relative expression levels of *Il17a*, *Il17f*, *Il21*, *Il22*, *Rorc*, *Rorα*, *Irf4*, and *Foxp3* were measured using SensiFAST Probe Hi-ROX kit (Bioline, Taunton, MA) and specific TaqMan probes (Applied Biosystems, Grand Island, NY). Samples were run on a StepOnePlus qPCR machine (Applied Biosystems). Results given are relative to actin control and wild type Th0 conditions (ΔΔC_T_). In some cases, wild type Th0 conditions were assigned a C_T_ value of 40 when no amplification occurred within 40 cycles.

### T cell transfer of colitis

Rag1-deficient mice were injected i.p. with approximately 3x10^5^ wild type or *Tpl2* ^*−/−*^ naïve T cells (CD4^+^CD25^−^CD45RB^hi^). Mice were weighed prior to injection and weekly thereafter. Blood was collected at 3, 6 and 8 weeks from the tail vein or by terminal cardiac puncture, and serum cytokines were quantified by Th1/Th2/Th17 cytokine bead array (BD Biosciences). Spleen and mesenteric lymph nodes were isolated and counted. Cells were restimulated *ex vivo* for 4 hours with PMA, ionomycin, and Golgi Plug (BD Biosciences) at a concentration of 1–2x10^6^ cells/ml and stained similarly to *in vitro* cultures.

### Pathology Scoring

Colonic sections from mice were collected and fixed in 10% neutral buffered formalin for 24 h at room temperature. Complete cross sections of formalin-fixed intestinal sections were placed in cassettes, embedded in paraffin, sectioned at 4 μm thickness, mounted on glass slides, and stained with hematoxylin and eosin (H&E). Histological sections were evaluated by a veterinary pathologist (TN) and scored according to the following criteria: (A) Distribution of the inflammation: 0 = None, 1 = Focal, 2 = Multifocal, 3 = Diffuse; (B) Degree of inflammation: 0 = None, 1 = Mild, 2 = Moderate, 3 = Severe; (C) Extent of erosion and/or ulceration: 0 = None, 1 = Superficial (lamina propria only), 2: Moderate (extends to the submucosa), 3: Severe (transmural) and then pooled to calculate total pathology score.

### Western Blotting

Cell pellets were washed in cold PBS and lysed in protein lysis buffer (dH_2_O, 0.05 M Tris, 0.3 M NaCl, 0.5% TTX 100, 2 mM EDTA, 0.4 mM Na_3_VO_4_, 2.5 mM Leupeptin, 2.5 mM Aprotinin, 2.5 mM 4-Nitrophenyl 4-guanidinobenzoate hydrochloride (NPGB)). Protein concentration was measured using a BCA protein assay (Thermo Scientific, Suwanee, GA). Twelve micrograms of total protein were separated on a 4–12% Bis-Tris gel (Life Technologies) and probed with antibodies for phospho-STAT3 (Ser727), phospho-STAT3 (Tyr705) and total STAT3 (Cell Signaling Technology, Danvers, MA).

### Statistics

*P* values were derived by paired or unpaired two-tailed Student’s t-test using Prism software, unless otherwise indicated. Differences were considered statistically significant if p≤0.05.

## Results

### Tpl2 promotes Th17 development *in vitro*

**Fig 1:**
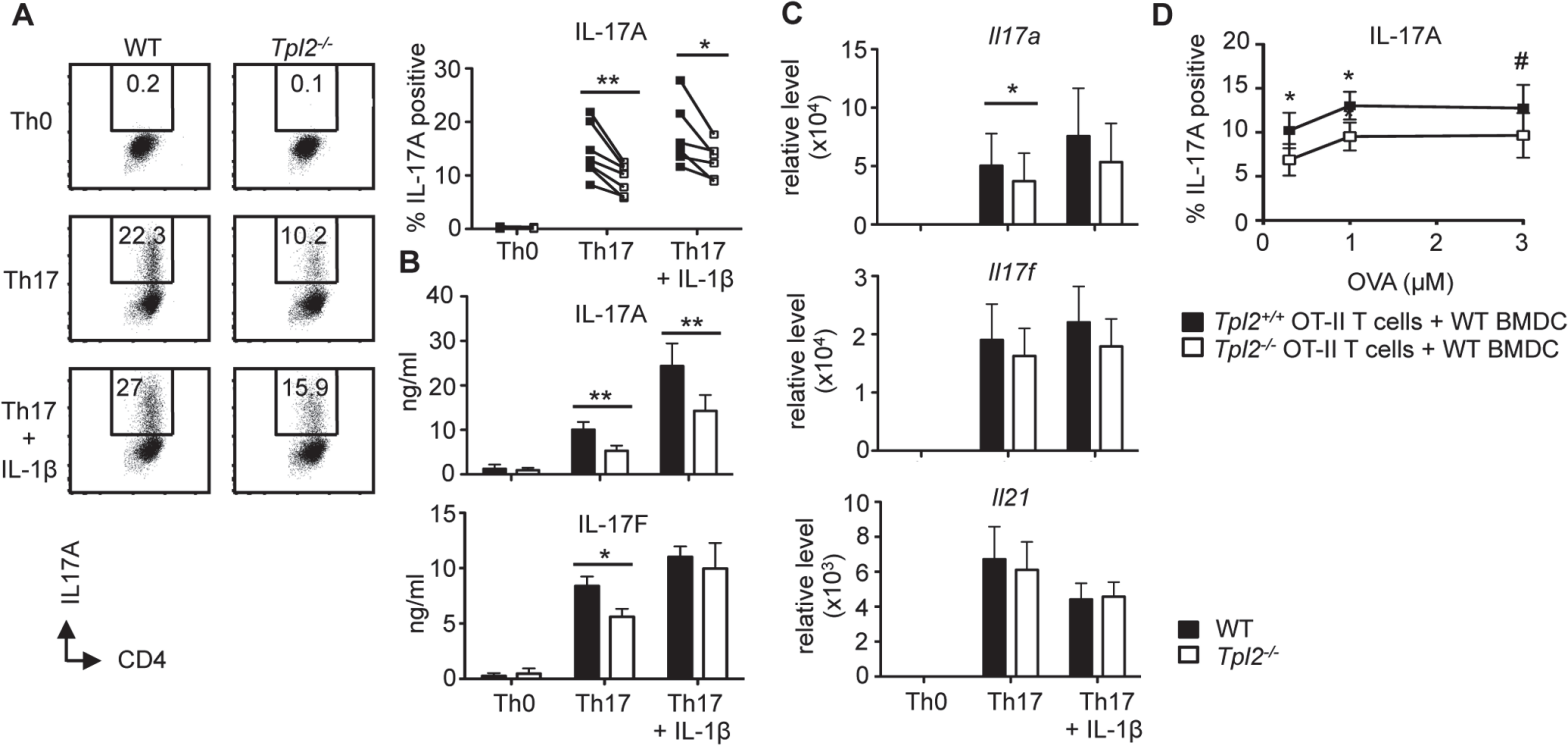
Tpl2 promotes Th17 development *in vitro* independently of IL-1β. Naïve T cells were cultured under Th17 polarizing conditions for 3 days +/− 10 ng/ml IL-1β. (A) IL-17A levels by flow cytometry in Th0 and Th17 conditions. Cells were gated on CD4^+^ cells. Connected symbols represent individual experiments. (B) IL-17A and IL-17F secretion by ELISA. (C) On day 3 of culture, *Il17a*, *Il17f*, and *Il21* expression levels by RT-PCR. (A-C) N≥6 experiments. (D) *Tpl2* ^*+/+*^ OT-II and *Tpl2* ^*−/−*^ OT-II naïve T cells were cultured for 3 days with wild type CD11c^+^ BMDCs, OVA peptide, and polarizing cytokines (IL-6 + TGF-β. IL-17A was measured by flow cytometry. Cells were gated on CD4^+^ cells. N = 3 experiments except where noted. #N = 2. Error bars represent means ± sem. *p<0.05, **p<0.005.

We previously demonstrated that Th17 differentiation was impaired in *Tpl2* ^*−/−*^ T cells [8]. To further confirm the importance of Tpl2 in Th17 differentiation *in vitro*, we stimulated wild type and *Tpl2* ^*−/−*^ naïve CD4 T cells in the presence of IL-6 and TGF-β. On day 3 of culture, *Tpl2* ^*−/−*^ CD4 T cells secreted significantly less IL-17A and IL-17F relative to wild type cells (Fig. 1A-B). Transcription of *Il17a* was also reduced in *Tpl2* ^*−/−*^ cells. No difference in transcription of *Il17f* or *Il21* was detected (Fig. 1C). Enhanced differentiation of wild type Th17 cells, as seen by increased production of IL-17A, was accomplished through addition of IL-1β to the cultures [30, 31]. Even with the addition of IL-1β, IL-17A production was still reduced in *Tpl2* ^*−/−*^ cells relative to wild type cells (Fig. 1A-B). Therefore, Tpl2 was required for optimal IL-17A production in CD4 T cells stimulated with IL-6 and TGF-β, and this defect could not be overcome by addition of exogenous IL-1β.

To confirm our results under more physiological conditions, we used a co-culture system to stimulate T cells with antigen presented in the context of MHC class II molecules by dendritic cells. We cultured either *Tpl2* ^*+/+*^ OT-II T cells or *Tpl2* ^*−/−*^ OT-II T cells expressing an OVA-specific transgenic TCR in the presence of their cognate antigen, ovalbumin peptide, with wild type BMDCs and the Th17 polarizing cytokines, IL-6 and TGF-β. Importantly, at all concentrations of OVA, *Tpl2* ^*−/−*^ Th17 cultures displayed reduced proportions of IL-17A positive cells (Fig. 1D). These results indicate that there is a T cell-intrinsic defect in the ability of *Tpl2* ^*−/−*^ Th17 cells to produce IL-17A.

### Tpl2 promotes Th1, but not Th17, differentiation in a CD45RB T cell transfer model of colitis

**Fig 2:**
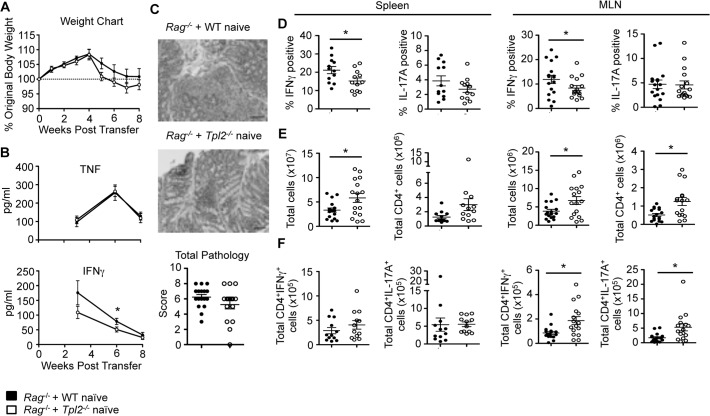
Tpl2 promotes Th1, but not Th17, differentiation in a CD45RB T cell transfer model of colitis. Wild type or *Tpl2* ^*−/−*^ naïve T cells (CD45RB^hi^CD25^-^CD4^+^) were injected i.p. into female Rag-deficient mice. (A) Weight loss curves as a percentage of original body weight. (B) Measure of serum cytokine levels in colitic mice. Significance was measured by one-tailed Student’s t-test. (C) Representative histologic images are shown along with scoring for total pathology in the colon. Pathology scores were evaluated using Mann-Whitney U test. (D) Proportions of IFNγ and IL-17A in the spleen and mesenteric lymph nodes as measured by intracellular staining and flow cytometry. Cells were gated on CD4^+^TCRβ^+^ cells. Significance was measured by one-tailed Student’s t-test. (E) Total cells and CD4^+^TCRβ^+^ cells in the spleen and mesenteric lymph nodes. (F) Total CD4^+^IFNγ^+^ and CD4^+^IL-17A^+^ cells in the spleen and mesenteric lymph nodes. N≥12. Pooled from 3 independent experiments. Error bars represent means ± sem. *p<0.05.

Having characterized a Tpl2-dependent defect in IL-17A expression, along with the previously identified IFNγ defect, we next examined the capacity of *Tpl2* ^*−/−*^ T cells to induce disease in a T cell transfer model of colitis associated with a mixed Th1 and Th17 inflammatory response. In this model, naïve CD4 effector T cells (CD4^+^CD25^-^CD45RB^hi^) adoptively transferred into Rag1-deficient mice undergo lymphopenia-induced expansion and cause intestinal inflammation that recapitulates human IBD [32]. Proliferating CD4 T cells respond to intestinal antigens to drive inflammation within the small and large intestines, characterized by increases in TNF, IFNγ, IL-17A, and IL-23, leading to weight loss and diarrhea [32–38]. When colitis was induced in Rag1-deficient mice, recipients of either wild type or *Tpl2* ^*−/−*^ naïve CD4 T cells experienced similar weight loss kinetics (Fig. 2A). As disease progressed, we observed increases in circulating TNF and IFNγ that waned at later time points once inflammation established within the intestine (Fig. 2B). Tpl2 ablation had no significant effect on the levels of circulating TNF at any time point but modestly reduced circulating IFNγ (Fig. 2B). Colitic mice were euthanized, and colons were scored for inflammation. Similar total pathology scores were observed between recipients of either wild type or *Tpl2* ^*−/−*^ cells (Fig. 2C).

Because of our previous identification of Tpl2 as a promoter of IFNγ secretion and Th1 differentiation [8], we hypothesized that Tpl2 ablation within the transferred T cell population would limit disease. Therefore, the nearly normal circulating IFNγ levels and colitis pathology were surprising. Since both Th1 and Th17 cells are associated with colitis, we investigated whether Tpl2 altered the proportions of IFNγ or IL-17A positive CD4 T cells within the spleen and mesenteric lymph nodes (MLN). Recipients of *Tpl2* ^*−/−*^ cells had reduced proportions of CD4^+^IFNγ^+^ cells, but not CD4^+^IL-17A^+^ cells (Fig. 2D). However, recipients of *Tpl2* ^*−/−*^ CD4 T cells also had more total cells and CD4 T cells within their spleens and MLN compared to recipients of wild type cells (Fig. 2E). Therefore, despite reduced proportions of CD4^+^IFNγ^+^ T cells within recipients of *Tpl2* ^*−/−*^ T cells, their absolute number was either unchanged or increased (depending on the organ) relative to recipients of wild type T cells (Fig. 2F).

### Tpl2 deficiency promotes FoxP3-mediated antagonism of IL-17A expression

**Fig 3:**
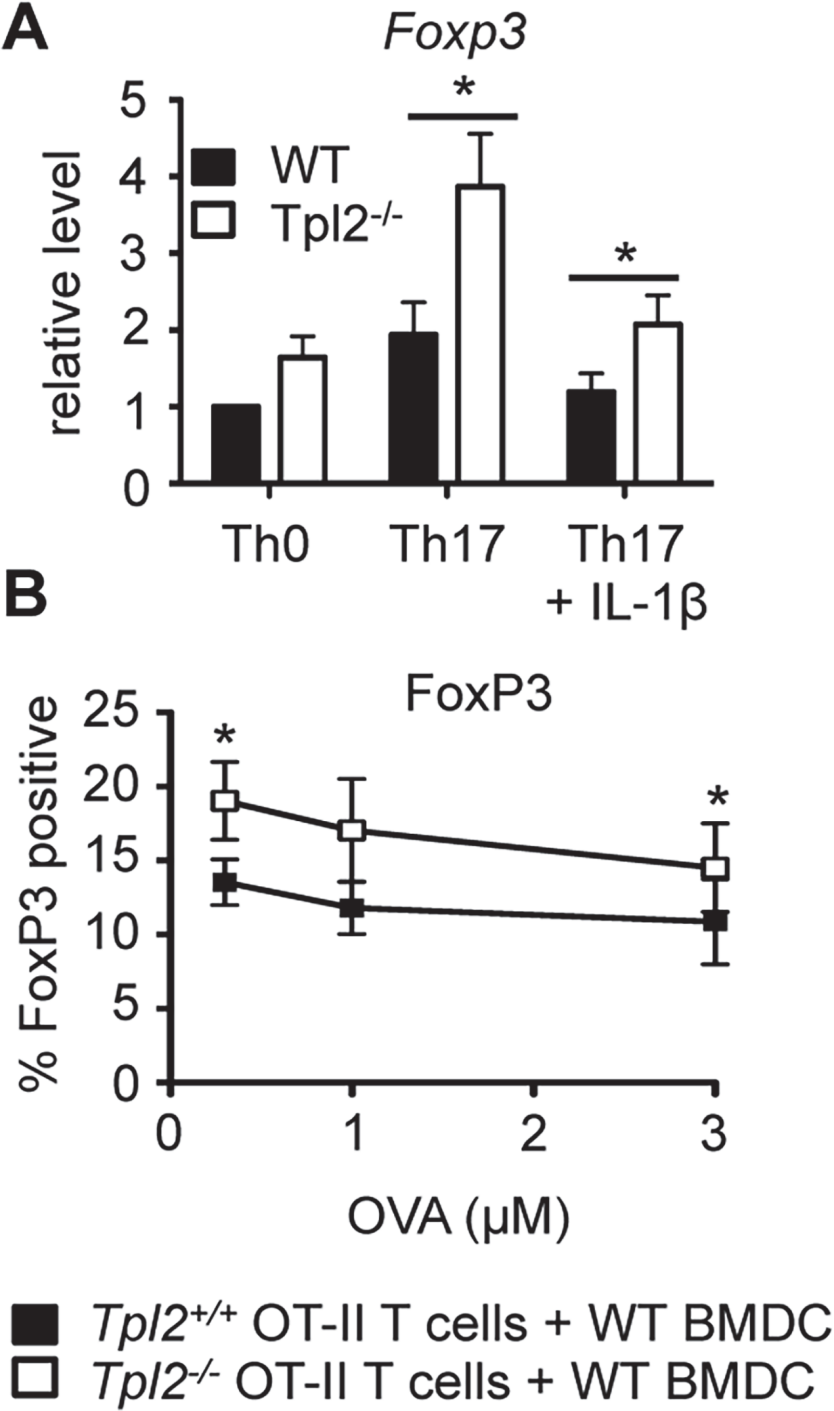
*Tpl2* ^*−/−*^ T cells express increased levels of the FoxP3 transcription factor. Naïve T cells were cultured under Th17 polarizing conditions for 3 days +/− 10 ng/ml IL-1β. (A) *Foxp3* expression was measured by RT-PCR. (B) *Tpl2* ^*+/+*^ OT-II and *Tpl2* ^*−/−*^ OT-II naïve T cells were cultured for 3 days with wild type CD11c^+^ BMDCs, OVA peptide, and polarizing cytokines (IL-6 + TGF-β). FoxP3 levels were measured by flow cytometry. Cells were gated on CD4^+^ cells. N = 3 experiments. Error bars represent means ± sem. *p<0.05.

In order to reconcile the conflicting requirements for Tpl2 in IL-17 production *in vitro* and *in vivo*, we next investigated the mechanism for reduced IL-17A production in *Tpl2* ^*−/−*^ T cells *in vitro* by determining whether reduced IL-17A production correlated with decreased levels of Th17-associated transcription factors. Th17-associated transcription factors include RORα, RORγt, BATF, IRF4, and STAT3 [39–42], but Th17 cell differentiation is negatively regulated by the transcription factor FoxP3 [43]. Surprisingly, in *Tpl2* ^*−/−*^ T cells, there was no impairment in *Rorc*, *Rorα*, *or Irf4* expression or STAT3 activation (S1 Fig.). In the absence of impaired expression or activation of transcription factors that induce IL-17A transcription, we reasoned that a negative regulatory mechanism was constraining IL-17A secretion in *Tpl2* ^*−/−*^ T cells. To examine whether FoxP3 expression was altered in *Tpl2* ^*−/−*^ T cells, we performed real-time PCR analysis of wild type and *Tpl2* ^*−/−*^ T cells cultured under Th17 conditions. *Tpl2* ^*−/−*^ Th17 cells expressed significantly elevated levels of FoxP3 relative to wild type Th17 cells (Fig. 3A). Additionally, in our co-culture system, *Tpl2* ^*−/−*^ Th17 cultures displayed higher proportions of FoxP3 positive cells compared to wild type (Fig. 3B) indicating there is a T cell-intrinsic defect in the ability of *Tpl2* ^*−/−*^ Th17 cells to produce IL-17A, which correlates with increased FoxP3 expression.

### Th17 differentiation is Tpl2-independent under conditions that fail to induce FoxP3

**Fig 4:**
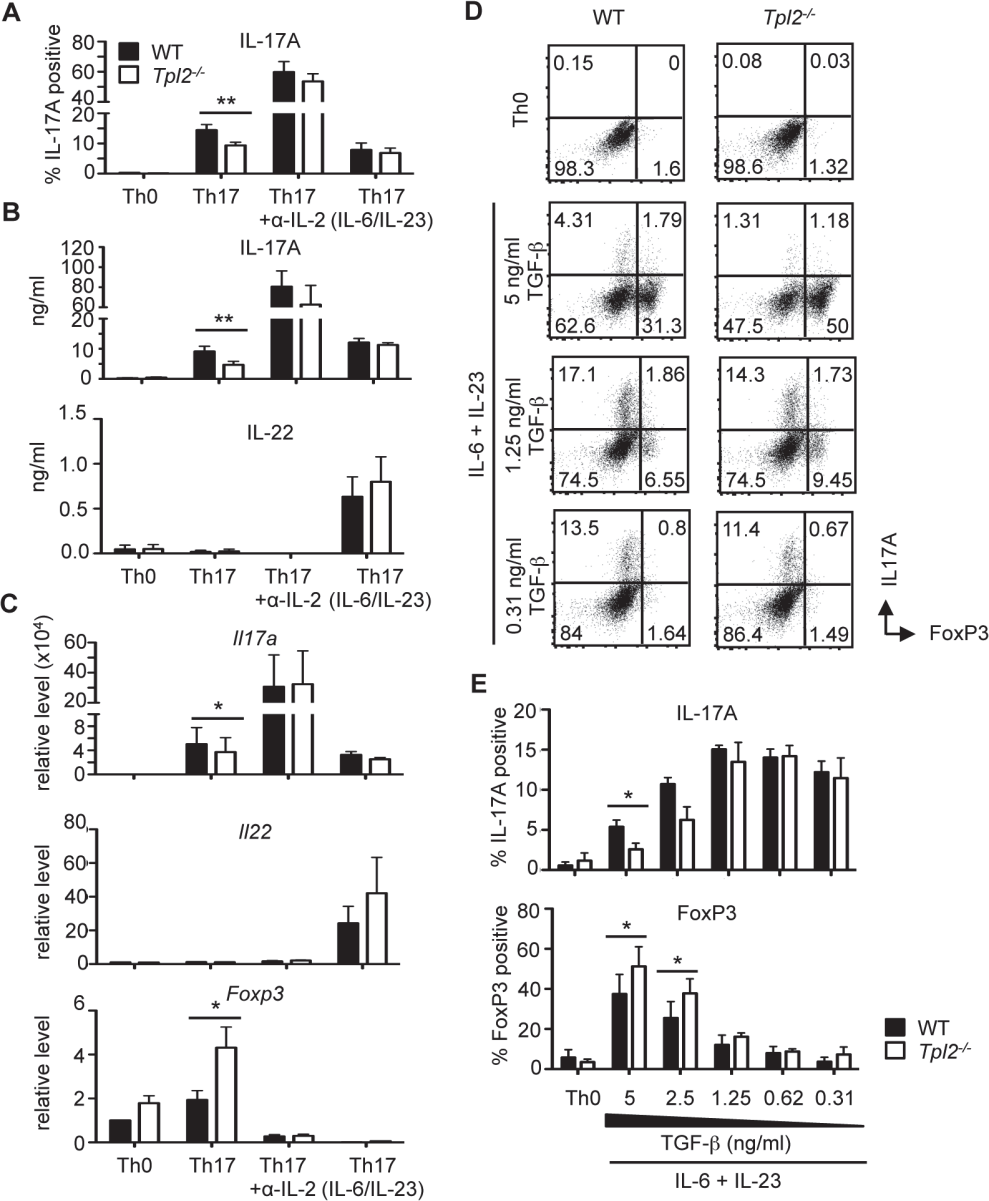
The differential regulation of IL-17A and FoxP3 by Tpl2 is TGF-β-dependent. Naïve T cells were cultured under Th17 polarizing conditions for 3 days +/− 5 μg/ml anti-IL-2 or with IL-6 and IL-23. (A) IL-17A levels were measured by intracellular staining and flow cytometry. Cells were gated on CD4^+^ cells. (B) IL-17A, IL-22, and IL-10 secretion was measured by ELISA. (C) *Il17a*, *Il22* and *Foxp3* expression was quantified by RT-PCR. (A-C) Pooled Th0 and Th17 results depicted in Figs. 1 and 2 are shown again for comparison. N≥3. (D) Representative flow plots of CD4^+^ cells gating on IL-17A and FoxP3 in Th0 and IL-6 + IL-23 + TGF-β (various concentrations) conditions. (E) Naïve T cells were cultured for 3 days with IL-6, IL-23, and varying concentrations of TGF-β. Proportions of IL-17A and FoxP3 positive cells were measured by intracellular staining and flow cytometry. Cells were gated on CD4^+^ cells. N = 3. Error bars represent means ± sem. *p<0.05, **p<0.005.

We next addressed whether Tpl2 was similarly dispensable for Th17 differentiation under alternative Th17-inducing conditions, some of which might more closely recapitulate the conditions present in the colitis model. First, we assessed the effect of IL-2, which inhibits Th17 differentiation [44] and promotes Treg cell differentiation [45], on Th17 differentiation of *Tpl2* ^*−/−*^ T cells. We added anti-IL-2 to cultures to neutralize its effects, and as expected, IL-2 neutralization reduced FoxP3 expression to below basal levels observed in Th0 conditions (Fig. 4C). Additionally, IL-2 neutralization significantly increased IL-17A differentiation, boosting the proportion of wild type IL-17A-expressing T cells from approximately 15% to 60% (Fig. 4A). Anti-IL-2 also greatly enhanced IL-17A expression and secretion (Fig. 4B-C). Notably, upon IL-2 neutralization, *Tpl2* ^*−/−*^ CD4^+^ T cells acquired the ability to produce IL-17A at wild type levels (Fig. 4A-C).

Because IL-2 neutralization reversed the phenotype, we investigated whether IL-2 secretion is increased in *Tpl2* ^*−/−*^ T cells. However, we observed no differences in IL-2 secretion between wild type and *Tpl2* ^*−/−*^ Th0 or Th17 cultures (S2 Fig.). Interestingly, IL-2 was nearly absent at this time in Th17 cultures (S2 Fig.). We next performed new experiments to observe IL-2 secretion over a time course under Th17 conditions. At days 1 and 2 of culture, wild type and *Tpl2* ^*−/−*^ T cells secreted similar levels of IL-2 (S2 Fig.). However, IL-2 was consumed within Th17 cultures by day 3, which matched the results from our initial day 3 cultures (S2 Fig.). Normal IL-2 secretion by *Tpl2* ^*−/−*^ T cells is consistent with a previous report [5]. These findings suggest that IL-2 secretion does not underlie the Th17 defect. Instead, they indicate that autocrine IL-2 permits TGF-β-induced FoxP3 expression, which is amplified in *Tpl2* ^*−/−*^ Th17 cells and suppress IL-17A expression.

In the absence of TGF-β, IL-6 and IL-23 have been shown to induce and expand Th17 cells that are more pathogenic and inflammatory *in vivo* than those driven by IL-6 and TGF-β, as they do not express FoxP3 [21, 46, 47]. We therefore cultured wild type and *Tpl2* ^*−/−*^ naïve T cells with IL-6 and IL-23. As expected, expression of *Foxp3* was extremely low in both wild type and *Tpl2* ^*−/−*^ Th17 cells cultured in this way (Fig. 4B-C). Under these conditions, *Tpl2* ^*−/−*^ Th17 cells produced wild type levels of IL-17A as seen by flow cytometry, ELISA, and RT-PCR (Fig. 4A-C). We also observed secretion and expression of IL-22 in Th17 cells cultured with IL-6 and IL-23 [24, 47], which was similar between wild type and *Tpl2* ^*−/−*^ cells (Fig. 4B-C). No significant IFNγ secretion was observed under these conditions (data not shown).

### The regulation of IL-17A and FoxP3 by Tpl2 is TGF-β-dependent

To confirm the importance of TGF-β-induced FoxP3 transcription in the reduction of IL-17A expression in *Tpl2* ^*−/−*^ T cells, we titrated TGF-β from Th17 conditions. For these studies, Th17 cells were induced by IL-6 and IL-23 in the presence of decreasing concentrations of TGF-β, and we assessed the effects on both IL-17A and FoxP3 induction. At high TGF-β concentrations, *Tpl2* ^*−/−*^ CD4 T cells expressed higher proportions of FoxP3 and reduced proportions of IL-17A compared to wild type cells (Fig. 4D-E). Littman *et al*. demonstrated that TGF-β, while required for optimal Th17 differentiation *in vitro*, could also suppress Th17 differentiation at high concentrations [43]. With reduced concentrations of TGF-β, proportions of IL-17A positive cells increased, proportions of FoxP3 positive cells dropped and both FoxP3 and IL-17A expression in *Tpl2* ^*−/−*^ cells normalized to wild type levels (Fig. 4E). Collectively, these findings demonstrate that there is no obligate requirement for Tpl2 in driving IL-17 or IL-22 secretion by CD4 T cells, but suggests instead that Tpl2 promotes TGF-β/FoxP3-mediated suppression of Th17 responses.

## Discussion

Herein, we describe a T cell-intrinsic defect in IL-17A production by *Tpl2* ^*−/−*^ Th17 cells driven by IL-6 and TGF-β. This defect in IL-17A production did not correlate with impaired expression of Th17 associated transcription factors RORα, RORγt, or IRF4 but was instead associated with increased levels of FoxP3. In the absence of FoxP3 induction, as seen with addition of anti-IL-2 to Th17 conditions or by alternatively differentiating Th17 cells with IL-6 and IL-23, there was no defect in IL-17A production by *Tpl2* ^*−/−*^ Th17 cells. Furthermore, titration of TGF-β revealed that increased FoxP3 expression and decreased IL-17A expression in *Tpl2* ^*−/−*^ cells were a direct consequence of TGF-β signaling. These findings demonstrate that Tpl2 normally constrains TGF-β-driven FoxP3 transcription, which allows for increased production of IL-17A.

A T cell transfer model of colitis was employed to directly assess the T cell-intrinsic functions of Tpl2 *in vivo* in driving autoimmune disease characterized by a mixed Th1 and Th17 pathology. Although Tpl2 promotes Th17 differentiation *in vitro*, Tpl2 did not alter Th17 differentiation in this model of colitis. There are several possible explanations for this. First, although it is standard practice to use TGF-β and IL-6 to drive Th17 differentiation *in vitro*, it has been well established that other factors are also important for Th17 development both *in vitro* and *in vivo*, such as IL-6 and IL-23. Th17 cells generated in this manner are more pathologic in a murine experimental autoimmune encephalomyelitis (EAE) model [46]. IL-23 is also required for disease development in the T cell transfer model of colitis [36]. The fact that Th17 differentiation induced by IL-6 and IL-23 was unaltered in *Tpl2* ^*−/−*^ T cells *in vitro* likely explains the normal Th17 differentiation in colitic recipients of *Tpl2* ^*−/−*^ effector cells. It remains possible that Th17 differentiation may be regulated by Tpl2 *in vivo* in a context-dependent manner where TGF-β concentrations are locally high, as at mucosal sites.

Interestingly, Tpl2 deficiency enhanced the lymphopenia-induced accumulation of transferred effector CD4 T cells in this colitis model. Lymphopenia-induced rapid proliferation occurs independently of IL-7 cytokine signals, but is thought to rely instead upon the TCR signal strength to available ligands within the lymphopenic host [33]. This raises the interesting possibility of either increased TCR signal strength within *Tpl2* ^*−/−*^ CD4 T cells that drives T cell proliferation or altered cell cycle progression in the absence of Tpl2. In this regard, Tpl2 ablation has been demonstrated to promote CD8 T cell proliferation in response to antigen stimulation [7]. In addition to T cells, *Tpl2* ^*−/−*^ colonic epithelial cells also proliferate at a higher rate with reduced apoptosis compared to wild type cells during DSS colitis, leading to tumor development [48]. Because increased cell survival has also been noted, we cannot exclude the possibility that increased accumulation of *Tpl2* ^*−/−*^ CD4 T cells may result from reduced apoptosis. Further studies are required to determine how Tpl2 affects CD4 T cell proliferation and survival *in vivo*. Despite significantly increased accumulation of *Tpl2* ^*−/−*^ effector cells within the lymphoid organs, recipients of *Tpl2* ^*−/−*^ T cells were no more susceptible to the development of colitis than recipients of wild type T cells. This was due to the impaired capacity of *Tpl2* ^*−/−*^ T cells to produce IFNγ. This finding is consistent with our previous work establishing Tpl2 as an important positive regulator of Th1 differentiation and IFNγ secretion [8].

The increased TGF-β-induced FoxP3 expression observed in *Tpl2* ^*−/−*^ T cells may have broader implications. In addition to its induction of Th17 cells, TGF-β is critical for the development and function of FoxP3^+^ immunosuppressive Tregs. Indeed, mice with mutations in TGF-β responsiveness, TGF-β secretion, or FoxP3 expression develop patent autoimmunity characterized by lymphoproliferation, cellular activation and pro-inflammatory cytokine secretion [49–53]. The observation that FoxP3 expression is increased in *Tpl2* ^*−/−*^ Th17 cells raises the possibility that FoxP3^+^ Tregs might also be increased in *Tpl2* ^*−/−*^ mice, further promoting the development of an immunosuppressive environment. If this is a generalized phenomenon, then Tpl2 inhibition would be expected to reduce severity of a range of autoimmune diseases where a more regulatory environment is desired. Ongoing studies are exploring this possibility.

Overall, our findings underscore the importance of Tpl2 in driving the development of the pro-inflammatory Th1 lineage. It further provides new insights into the regulation of TGF-β-induced FoxP3 expression as well as lymphopenia-induced expansion of T cells by Tpl2. These findings support the use of Tpl2 inhibitors for the targeted treatment of Th1-driven autoimmune diseases, such as diabetes and colitis [38, 54, 55] but suggest that Tpl2 inhibitors may have more limited utility in treating Th17-mediated diseases. Further studies are needed to fully elucidate the effects of Tpl2 in specific autoimmune disease settings on a case-by-case basis, as the specific cytokine milieu will differentially engage the Tpl2 kinase.

## Supporting Information

S1 Fig Tpl2 does not regulate Th17 transcription factor expression or activation. Naïve T cells were cultured under Th17 polarizing conditions for 3 days +/- 10 ng/ml IL-1β. (A) *Rorc*, *Rora* and *Irf4* expression was measured by RT-PCR on day 3 of culture. N≥6 experiments. (B) Th0 cells cultured for 3 days were expanded an additional 4 days with IL-2 (40 IU/ml) prior to stimulation with IL-6 (10 ng/ml) for 30 minutes at 37°C. Whole cell lysates were immunoblotted for phosphorylated STAT3 (pSTAT3) and total STAT3 (STAT3). N = 2 experiments. (C) *Il23r* expression by RT-PCR of T cells cultured for up to 3 days +/- 10 ng/mL IL-6. Expression levels are relative to wild type day 0. N = 2 experiments.
(TIF)

S2 Fig Tpl2 does not regulate IL-2 secretion in Th0 or Th17 cells. Naïve CD4 T cells were cultured under Th17 polarizing conditions or with IL-6 and IL-23 up to 3 days. (A) On day 3, supernatants were collected and analyzed for IL-17A and IL-2 secretion by ELISA. Data shown are representative of 4 independent experiments. (B) On days 1 through 3, supernatants were collected from Th17 cultures and analyzed for IL-2 secretion by ELISA. N≥4. Error bars represent means ± SE. *p<0.05
(TIF)
