# Supplementary figures and images for: Tumor Progression Locus 2 Differentially Regulates IFNγ and IL-17 Production by Effector CD4+ T Cells in a T Cell Transfer Model of Colitis
Source: PLoS One. 2015 Mar 17;10(3):e0119885. doi: 10.1371/journal.pone.0119885 (PMC4363566; doi:10.1371/journal.pone.0119885)

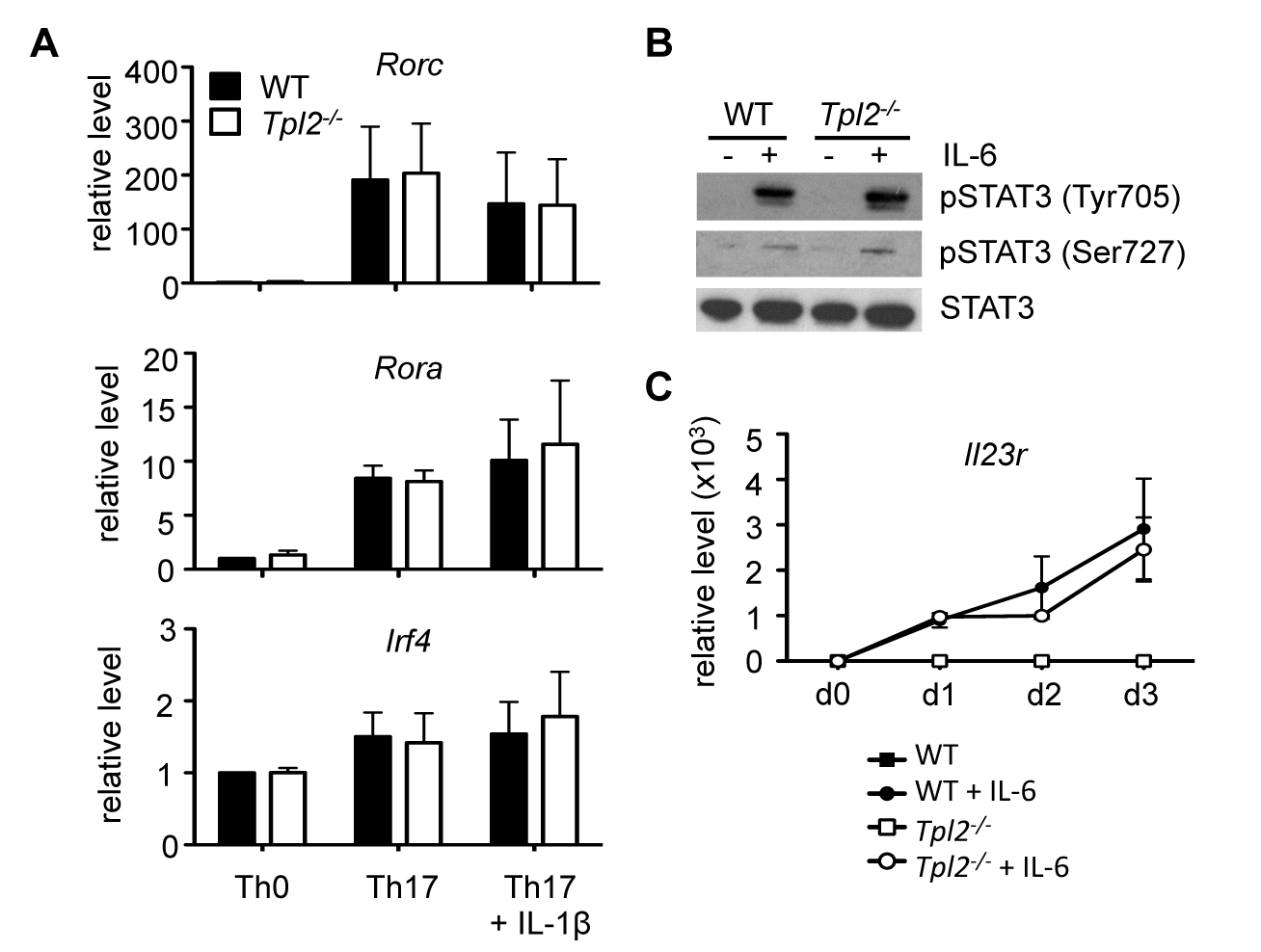

Supplement: S1 Fig — Naïve T cells were cultured under Th17 polarizing conditions for 3 days +/- 10 ng/ml IL-1β. (A) Rorc, Rora and Irf4 expression was measured by RT-PCR on day 3 of culture. N≥6 experiments. (B) Th0 cells cultured for 3 days were expanded an additional 4 days with IL-2 (40 IU/ml) prior to stimulation with IL-6 (10 ng/ml) for 30 minutes at 37°C. Whole cell lysates were immunoblotted for phosphorylated STAT3 (pSTAT3) and total STAT3 (STAT3). N = 2 experiments. (C) Il23r expression by RT-PCR of T cells cultured for up to 3 days +/- 10 ng/mL IL-6. Expression levels are relative to wild type day 0. N = 2 experiments. (TIF) [file pone.0119885.s001.tif]

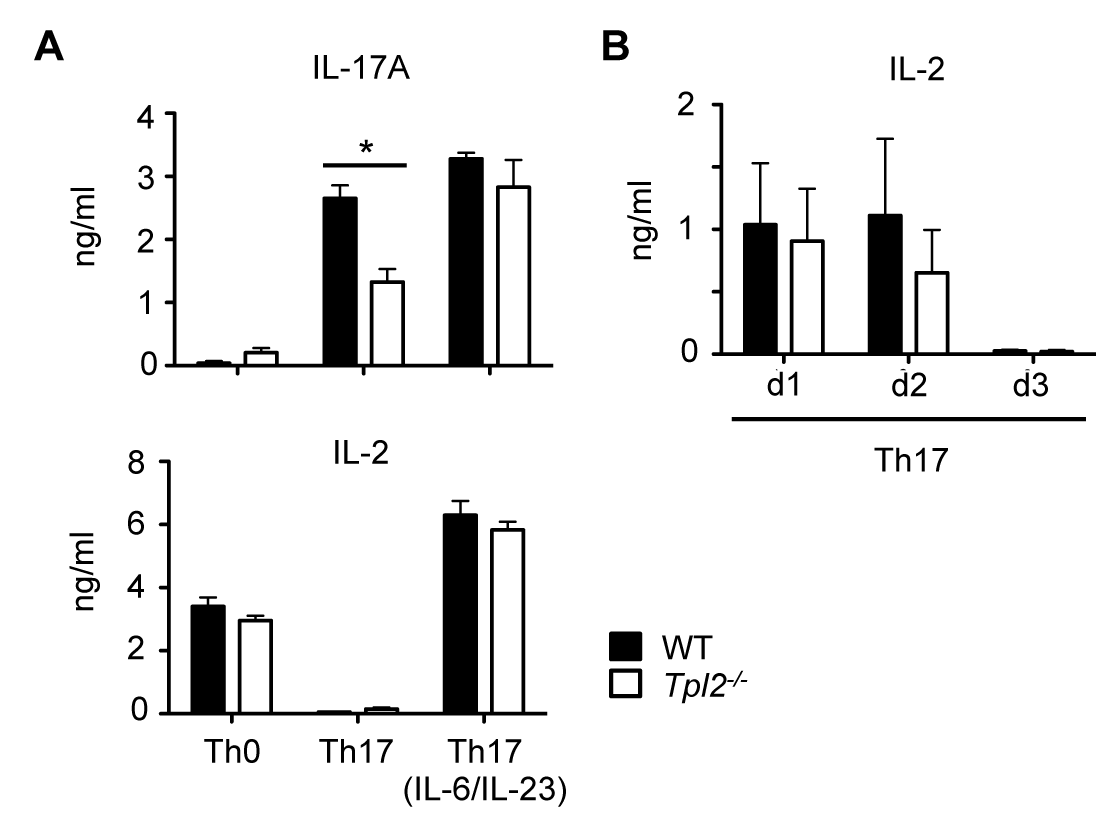

Supplement: S2 Fig — Naïve CD4 T cells were cultured under Th17 polarizing conditions or with IL-6 and IL-23 up to 3 days. (A) On day 3, supernatants were collected and analyzed for IL-17A and IL-2 secretion by ELISA. Data shown are representative of 4 independent experiments. (B) On days 1 through 3, supernatants were collected from Th17 cultures and analyzed for IL-2 secretion by ELISA. N≥4. Error bars represent means ± SE. *p<0.05 (TIF) [file pone.0119885.s002.tif]
